# Soluble Tumor Necrosis Factor Receptor 2: A Promising Predictive Biomarker for Renal Dysfunction in Membranous Glomerulonephritis

**DOI:** 10.7759/cureus.58506

**Published:** 2024-04-18

**Authors:** Srinivas Nagaram, Priscilla Charles, Hanumanthappa Nandeesha, Norton Stephen, Sreejith Parameswaran, Palanivel Chinnakali, Rajesh Nachiappa Ganesh

**Affiliations:** 1 Department of Pathology, Jawaharlal Institute of Postgraduate Medical Education and Research, Puducherry, IND; 2 Department of Biochemistry, Jawaharlal Institute of Postgraduate Medical Education and Research, Puducherry, IND; 3 Department of Nephrology, Jawaharlal Institute of Postgraduate Medical Education and Research, Puducherry, IND; 4 Department of Preventive Medicine, Jawaharlal Institute of Postgraduate Medical Education and Research, Puducherry, IND

**Keywords:** tumor necrosis factor alpha, renal failure, membranous glomerulonephritis, estimated glomerular filtration rate, chronic kidney disease

## Abstract

Background and objective

Membranous glomerulonephritis (MGN) is a common cause of adult nephrotic syndrome. Tumor necrosis factor-α (TNF-α) is a proinflammatory cytokine that signals by attaching to TNF receptors. TNF-α plays a pivotal role in the development and progression of different forms of glomerulonephritis. Several research findings suggest that TNF-α receptors (TNFR1 and TNFR2) are predictors of estimated glomerular filtration rate (eGFR) decline. In light of this, this study aimed to explore the relationship between TNFR2 and eGFR, as well as the predictive role of TNFR2 in eGFR decline in MGN.

Methods

A total of 50 consecutive patients with a diagnosis of primary MGN based on renal biopsies and clinical workups were included in the study. TNFR2 levels in serum, urine, and gene expression were evaluated at baseline and after three months of follow-up by using enzyme-linked immunosorbent assay (ELISA) kits for TNFR2 (KTE60215, Abbkine, Wuhan, China). Cox regression was employed to determine the predictive significance of TNFR2 in persistent eGFR decline. Additionally, an ROC curve analysis was conducted to assess the prognostic value of TNFR2 in predicting persistent eGFR decline among MGN patients.

Results

We assessed the levels of inflammatory markers TNF-α and TNFR2, examined their correlation with eGFR and renal injury, and investigated their potential in predicting persistent eGFR. Patients with MGN exhibited elevated levels of TNFR2 in their serum, urine, and gene expression compared to healthy individuals. Additionally, there was a positive correlation between serum TNFR2 and TNF-α, urine protein-creatinine ratio (UPCR), uric acid, and total cholesterol. Conversely, there was a negative correlation with eGFR, serum albumin, and calcium. Serum TNFR2 showed statistical significance in a univariate Cox regression analysis (HR: 1.010, 95% CI: 1.00-1.01, p = 0.045) for predicting a persistent decline in eGFR. However, it did not show significance concerning relapse and remission. An ROC curve was created to assess TNFR2's prognostic potential as a biomarker, demonstrating an AUC of 0.683, with a sensitivity of 68% and specificity of 64%.

Conclusions

Based on our findings, TNFR2 is a predictive biomarker for eGFR decline in MGN, correlating with renal inflammation and predicting deterioration in renal function. TNFR2 emerges as a promising biomarker for early identification in patients at risk of renal function decline.

## Introduction

Membranous glomerulonephritis (MGN) is an autoimmune condition where the immune system produces autoantibodies targeting podocyte antigens such as M-type phospholipase A and thrombospondin 7A [1]. The majority of glomerulonephritis cases are characterized by the deposition or formation of immune complexes within the glomeruli, triggering both humoral and cellular immune responses. This leads to proteinuria and a decline in renal function due to glomerular injury [2]. Kidney failure is common among individuals with glomerulonephritis, primarily due to an influx of activated inflammatory cells in the glomeruli, leading to injury and damage to interstitial tissue, subsequently resulting in fibrosis. Aggressive immunosuppressive therapy involving corticosteroids and cyclophosphamide has significantly enhanced the outcome for most patients with MGN. However, a minority of patients may not respond well to immunosuppressive treatments despite receiving the standard of care for glomerulonephritis [3].

Various studies have indicated that the tumor necrosis factor (TNF) pathway plays a role in the development of different renal diseases, and TNF-related biomarkers are also linked with levels of albuminuria or glomerular filtration rate (GFR) [4]. Tumor necrosis factor alpha (TNF-α) is a proinflammatory cytokine generated by macrophages, T cells, and various kidney cells. It is also expressed by neutrophils, mast cells, and endothelial cells [2,5]. It exists in two forms: soluble and transmembrane. Initially synthesized as a precursor, it undergoes processing by TNF-α-converting enzyme (TACE) to be released as soluble TNF-α (sTNF-α). Upon binding to tumor necrosis factor receptors (TNFRs) [tumor necrosis factor receptor 1 (TNFR1) and tumor necrosis factor receptor 2 (TNFR2)], TNF-α transmits molecular signals regulating biological functions such as inflammation and cell death [6]. TNF-α and its receptors function in an independent manner from other commonly studied biomarkers in MGN such as anti-M type phospholipase A2 receptor (anti-PLA2R), retinol-binding protein, beta 2 microglobulin, several novel podocyte antigens and their antibodies, novel metabolite and protein targets, immune cells, non-coding RNA, etc. We focus on TNFR2 in our study given the availability of novel therapeutic targets potentially enabling personalized targeted therapy.

Inflammation has been recognized as a significant factor contributing to scarring and advancement of chronic kidney disease (CKD), irrespective of the cause. Current findings indicate a correlation between inflammatory biomarkers and the decline in kidney function, reflecting the underlying mechanisms of kidney injury [7]. MGN results in elevated expression of inflammatory mediators, including key proinflammatory cytokines such as interleukin-1 beta (IL-1β), interleukin-6 (IL-6), and TNF-α. These cytokines are pivotal in driving the progression of MGN [8]. Intrinsic kidney cells are the predominant cellular source of TNF, leading to inflammatory damage in glomerulonephritis, while invading leukocytes do not contribute [9]. In normal kidneys, TNFR1 showed consistent expression, while TNFR2 was notably absent. However, in cases of allograft rejection, TNFR2 was upregulated particularly in tubular epithelial cells. TNFR2 is primarily situated on the cell membrane in cultured cells, whereas TNFR1 is predominantly localized within the Golgi apparatus, with only a limited number of receptors observable on the cellular surface [10]. TACE facilitates the release of TNFR1 and TNFR2 from the cell membrane through shedding [11].

Evidence suggests that circulating TNF-α receptors correlate positively with urinary albumin-to-creatinine ratio (ACR) and negatively with estimated glomerular filtration rate (eGFR) in patients with various renal disorders. [12] These biomarkers also predict not only a decrease in GFR over time but also an increased risk of mortality for individuals with diabetes and other kidney ailments [4,13]. High levels of circulating TNFRs (cTNFRs) upon diagnosis served as effective indicators for faster decline of renal function. Nonetheless, the specific mechanisms behind elevated cTNFR levels linked to a heightened risk of renal progression remain uncertain [14]. This study aims to assess the predictive value of TNFR2 in estimating the persistent decline of eGFR in patients diagnosed with MGN.

## Materials and methods

Study design

This study aimed to explore the association between serum and urine levels of TNFR2 and their predictive and prognostic significance regarding the decline in eGFR and relapse and remission in patients with MGN. It was conducted at the Department of Pathology, Jawaharlal Institute of Medical Education and Research (JIPMER), after obtaining approval from the JIPMER ethics committee (JIP/IEC/2019/070); it adhered to the ethical principles outlined in the Declaration of Helsinki.

Characteristics of study participants

After obtaining informed consent, we enrolled 50 patients with newly diagnosed nephrotic syndrome from the nephrology department, with biopsy-proven MGN. Patients with obvious malignancies, secondary causes such as lupus, and the use of incriminating drugs were excluded. Patients were followed up for 18 months. The baseline clinical characteristics of each patient were documented. Blood and urine samples were obtained for laboratory analysis. A 2 ml blood sample was collected in a serum separator tube, centrifuged, and stored at -80 °C until analysis. Additionally, 2 ml of blood collected in an EDTA tube was utilized for RNA isolation. Samples were collected twice: initially upon enrolment and again at the three-month follow-up to assess serum and urine TNFR2 levels and gene expression. Tissue biopsy samples were obtained from patients primarily for diagnostic purposes, followed by immunohistochemical (IHC) analysis to evaluate TNFR2 expression. The eGFR was calculated using the CKD-EPI creatinine 2009 equation and the National Kidney Foundation application software. Remission and relapse were defined per the 2021 KDIGO (Kidney Disease: Improving Global Outcomes) Clinical Practice Guideline for Managing Glomerular Diseases [15,16]. Additionally, blood and urine samples were collected from an equal number of age- and sex-matched healthy controls to establish correlations between observed levels in patients and those in the control group.

Measurement of TNFR2 and TNF-α

Serum and urine samples were thawed and subjected to protein quantification using enzyme-linked immunosorbent assay (ELISA) kits for TNFR2 (KTE60215, Abbkine, Wuhan, China) and TNF-α (KTE6032, Abbkine, Wuhan, China). A Bio-Rad washer and a microplate reader were utilized in the process. The sandwich ELISA technique was employed, with 10 µl of sample used for TNFR2 quantification and 100 µl for TNF-α quantification. The optical density (OD) of each well was read within 15 minutes by using a microplate reader set to 450 nm. The concentration of the proteins was determined by using a standard curve generated using the kit's standards.

Gene expression

RNA was isolated from peripheral blood using the QIAamp RNA Blood Mini Kit (52304, QIAGEN, Hilden, Germany) and eluted in 100 μl of nuclease-free water. The isolated RNA was stored at -80 °C until further processing. To ensure the absence of protein and DNA contamination, RNA purity was assessed using a Qubit™ RNA HS Assay Kit (Q32852, Invitrogen by Thermo Fisher Scientific, Waltham, MA) with the Qubit 3.0 Fluorometer (Q33216, Invitrogen by Thermo Fisher Scientific). Subsequently, contaminant-free RNA was converted into complementary DNA (cDNA) using the high-capacity cDNA conversion Kit (4368814, Applied Biosystems by Thermo Fisher Scientific). The cDNA was then amplified and quantified in a 20 µl PCR reaction utilizing the Applied Biosystems QuantStudio 3 Thermal Cycler and TaqMan Fast Advanced PCR Master Mix (4444963, Applied Biosystems by Thermo Fisher Scientific). TaqMan probes specific for the target genes TNFR2 (Hs00153550_m1, Applied Biosystems by Thermo Fisher Scientific) and the housekeeping gene GAPDH (Hs99999905_m1, Applied Biosystems by Thermo Fisher Scientific) were employed for this purpose. The first two steps of PCR amplification were UNG incubation at 50°C for two minutes and Taq polymerase activation at 95 °C for two minutes, followed by 40 cycles of denaturation at 95°C for one second and annealing at 60°C for 20 seconds. The samples were analyzed using triplicate runs for each probe, and the mean value was utilized for the analysis. Relative quantification was performed for all targets to determine the fold change using the formula 2^-ΔΔCt [17].

Immunohistochemical analysis

Formalin-fixed paraffin sections of embedded renal tissue were cut to a thickness of 3 to 4 microns. The tissue sections underwent deparaffinization and rehydration, followed by the inhibition of endogenous peroxidase activity by using 5% hydrogen peroxide (H_2_O_2_). Antigen retrieval was accomplished by using a pressure cooker and citrate buffer, followed by washing with Tris-buffered saline (TBS). The slides were incubated overnight at 4 °C with the rabbit primary antibody [anti-TNF receptor II antibody (EPR1653), Abcam, Cambridge, UK]. Following incubation, the slides were washed with TBS. Afterward, the slides were incubated for 40 minutes with a secondary horseradish peroxidase (HRP) antibody. Slides were washed again after incubation with the secondary antibody. To visualize the antigen-antibody complexes, DAB (3,3'-diaminobenzidine) chromogen was applied and incubated for five minutes. The slides were cleaned with distilled water. Harris hematoxylin was used as a counterstain. Primary antibodies were used at the following dilutions: TNFR2 (1:400). The H-scoring system was used to determine the proportion and intensity of marker expression in the podocytes, proximal convoluted tubules (PCT), and distal convoluted tubules (DCT).

Statistical analysis

The Shapiro-Wilk test was utilized to assess the distribution of the data. Continuous variables were summarized using either mean ± standard deviation (SD) or median with interquartile range (IQR, 25th and 75th percentile), while categorical variables were summarized using percentages. Bivariate Spearman rank correlation was employed to investigate the relationships between serum and urine TNFR2 and various factors including age, MAP, eGFR, UPCR, TNF-α, other biochemical parameters, and histopathological findings. The study employed the Wilcoxon signed-rank test to compare the baseline and follow-up groups, while the Mann-Whitney U test was utilized for comparing cases with healthy controls. Time-to-event analysis along with endpoint assessment utilized Kaplan-Meier analysis in conjunction with a log-rank test. Cox regression analysis was used to identify potential predictors of outcomes. An ROC curve assessed predictive utility for the persistent decline in eGFR <30 ml/kg/min/1.73m^2^. SPSS Statistics version 19 (IBM Corp., Armonk, NY) and GraphPad Prism version 8.0.2 (Insight Partners, New York City, NY) were used for all analyses, and a p-value <0.050 was considered statistically significant.

## Results

The baseline demographic and biochemical characteristics of the study participants are presented based on the median TNFR2 level (Table 1).

**Table 1:** Demographic and biochemical parameters according to median serum TNFR2 level SD: standard deviation; IQR: interquartile range; TNFR2: tumor necrosis factor receptor 2; MAP: mean arterial pressure; eGFR: estimated glomerular filtration rate; UPCR: urine protein-to-creatinine ratio; TNF-α: tumor necrosis factor-alpha; AST: aspartate aminotransferase; ALT: alanine aminotransferase; ALP: alkaline phosphatase; WBC: White blood cell count

Parameter	Total	Above median (>358.62)	Below median (<358.62)	P-value
Age, years, mean ± SD	41.9 ± 13.3	40.4 ± 14.6	43.4 ± 12.5	0.353
MAP, mmHg, mean ± SD	94.49 ± 13.13	95.45 ± 12.84	92.08 ± 11.68	0.313
eGFR, mL/min/1.73 m², median (IQR)	96.5 (47.5 – 114)	60.5 (30.5 – 111.2)	105 (90 – 114.7)	0.039
UPCR, mg/mg, median (IQR)	3.7 (2.1 – 6.62)	6.5 (3.7 – 9.8)	2.1 (0.90 – 3.7)	0.001
TNF-α, pg/mL, mean ± SD	16.33 ± 8.13	18.68 ± 8.23	13.99 ± 7.48	0.021
Serum albumin, gm/dL, mean ± SD	2.94 ± 0.85	2.58 ± 0.79	3.30 ± 0.77	0.001
Total protein, gm/dl, mean ± SD	5.54 ± 1	5.12 ± 0.89	5.96 ± 0.93	0.001
Blood glucose, mg/dL, median (IQR)	106.42 (92.57 – 122.74)	101.66 (90.25 – 118.25)	106.5 (93.52 – 129.12)	0.288
Blood urea, mg/dL, median (IQR)	30.89 (22.18 – 42.13)	36.66 (23.70 – 57.61)	26.33 (22.02 – 36.22)	0.007
Sodium, mEq/L, median (IQR)	138.11 (136.53 – 139.08)	137.60 (136.35 – 139.43)	138.17 (136.5 – 139.62)	0.886
Potassium, mEq/L, mean ± SD	4.23 ± 0.41	4.20 ± 0.39	4.25 ± 0.43	0.755
Calcium, mg/dL, mean ± SD	8.16 ± 1.84	7.87 ± 1.79	8.46 ± 1.88	0.279
Magnesium, mg/dL, median (IQR)	1.96 (1.86 – 2.11)	2.03 (1.86 – 2.14)	1.95 (1.84 – 2.10)	0.247
Phosphorus, mg/dL, mean ± SD	4.07 ± 0.53	4.04 ± 0.53	4.12 ± 0.58	0.637
Chloride, mEq/L, mean ± SD	103.79 ± 4.79	103.36 ± 5.34	103.95 ± 4.55	0.671
Uric acid, mg/dL, mean ± SD	6.32 ± 1.89	6.57 ± 1.54	5.95 ± 2.15	0.240
Total bilirubin, mg/dL, median (IQR)	0.41 (0.29 – 0.56)	0.39 (0.28 – 0.50)	0.45 (0.30 – 0.64)	0.279
Direct bilirubin, mg/dL, median (IQR)	0.07 (0.05 – 0.11)	0.06 (0.05 – 0.07)	0.09 (0.05 – 0.14)	0.237
AST, IU/L, median (IQR)	23.22 (18.67 – 26.83)	25.21 (17.08 – 31.40)	21.08 (18.81 – 26.10)	0.700
ALT, IU/L, median (IQR)	20.25 (13 – 27.70)	20.66 (11.75 – 28.53)	20.22 (15.20 – 25.68)	0.458
ALP, IU/L, median (IQR)	92.13 (67.82 – 110.91)	102 (64.01 – 111.66)	89.21 (78.66 – 110.50)	0.456
Total cholesterol, mg/dL, median (IQR)	280.67 (205.17 – 328.67)	306.66 (274.12 – 376.31)	265 (168.25 – 300.25)	0.030
Hemoglobin, gm/dL, median (IQR)	11.07 (9.60 – 12.11)	10.80 (9.36 – 11.80)	11.61 (10.24 – 13.02)	0.130
WBC count, cells/μL, mean ± SD	9.0 ± 1.84	8.89 ± 1.76	9.06 ± 1.97	0.769

**Figure 1:**
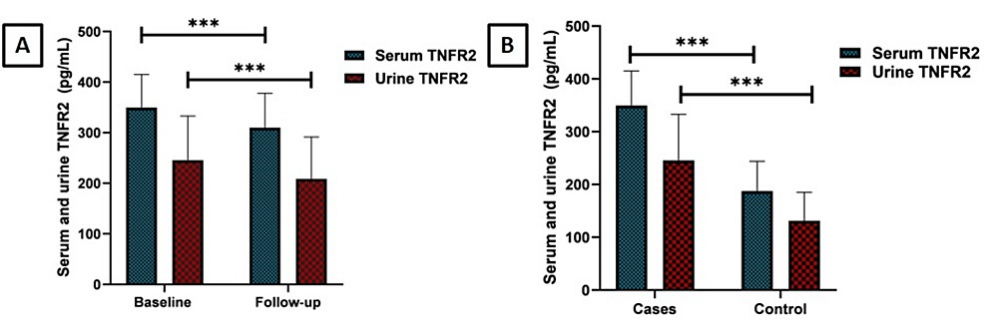
Comparison of serum and urine TNFR2 levels between cases at baseline and follow-up, and between cases and controls TNFR2: tumor necrosis factor receptor 2

The average age for men was 44.77 ± 13.69 years, while it was 34.57 ± 9.31 years for women. Serum and urine TNFR2 levels were significantly higher in patients compared to controls, and there was a significant difference between baseline and follow-up serum and urine TNFR2 levels (Figure 1).

Serum TNFR2 levels were positively correlated with UPCR (r = 0.731, p<0.001), serum TNF-α (r = 0.380, p = 0.006), total cholesterol (r = 0.356, p = 0.024), and uric acid (r = 0.327, p = 0.023), while they negatively correlated with eGFR (r = -0.409, p = 0.004), serum albumin (r = -0.397, p = 0.004), and calcium (r = -0.349, p = 0.013). Urine TNFR2 showed a negative correlation with eGFR (r = -0.552, p<0.001), and serum albumin (r = -0.439, p<0.001), but a positive correlation with UPCR (r = 0.709, p<0.001) and TNF-α (0.343, p = 0.015) (Table 2).

**Table 2:** Correlation of serum TNFR2 levels with age, MAP, eGFR, and other biochemical parameters Alb: albumin; Ca: calcium; eGFR: estimated glomerular filtration rate; MAP: mean arterial pressure; P: phosphorus; TC: total cholesterol; TNF-α: tumor necrosis factor-alpha; UPCR: urine protein creatinine ratio; UA: uric acid

		Age	MAP	eGFR	UPCR	TNF-α	Alb	Ca	P	UA	TC	WBC
Serum TNFR2	r	-0.037	-0.044	-0.409	0.731	0.380	-0.397	-0.349	0.188	0.327	0.356	-0.014
	p-value	0.799	0.766	0.004	<0.001	0.006	0.004	0.013	0.201	0.023	0.024	0.923
Age	r	1.000	0.119	-0.421	0.154	0.095	-0.087	-0.058	-0.133	-0.036	-0.005	0.141
	p-value	-	0.413	0.003	0.307	0.511	0.548	0.689	0.367	0.810	0.973	0.335
MAP	r	-	1.000	-0.209	0.068	0.155	-0.001	0.093	0.011	0.224	-0.031	-0.054
	p-value	-	-	0.158	0.655	0.287	0.993	0.526	0.940	0.130	0.851	0.715
eGFR	r	-	-	1.000	-0.238	-0.376	-0.018	-0.009	-0.028	-0.308	-0.041	0.104
	p-value	-	-	-	0.120	0.008	0.903	0.953	0.852	0.037	0.804	0.487
UPCR	r	-	-	-	1.000	0.319	-0.573	-0.510	0.201	0.120	0.426	0.265
	p-value	-	-	-	-	0.030	<0.001	<0.001	0.185	0.432	0.008	0.075
TNF-α	r	-	-	-	-	1.000	-0.124	-0.073	0.066	0.232	0.200	0.191
	p-value	-	-	-	-	-	0.391	0.616	0.655	0.113	0.216	0.188
SA	r	-	-	-	-	-	1.000	0.909	-0.119	0.019	-0.456	-0.246
	p-value	-	-	-	-	-	-	<0.001	0.420	0.898	0.003	0.089
Ca	r	-	-	-	-	-	-	1.000	-0.093	0.044	-0.417	-0.147
	p-value	-	-	-	-	-	-	-	0.528	0.767	0.007	0.313
P	r	-	-	-	-	-	-	-	1.000	0.084	0.012	0.073
	p-value	-	-	-	-	-	-	-	-	0.569	0.942	0.625
UA	r	-	-	-	-	-	-	-	-	1.000	0.061	0.245
	p-value	-	-	-	-	-	-	-	-	-	0.711	0.097
TC	r	-	-	-	-	-	-	-	-	-	1.000	0.201
	p-value	-	-	-	-	-	-	-	-	-	-	0.219

**Figure 2:**
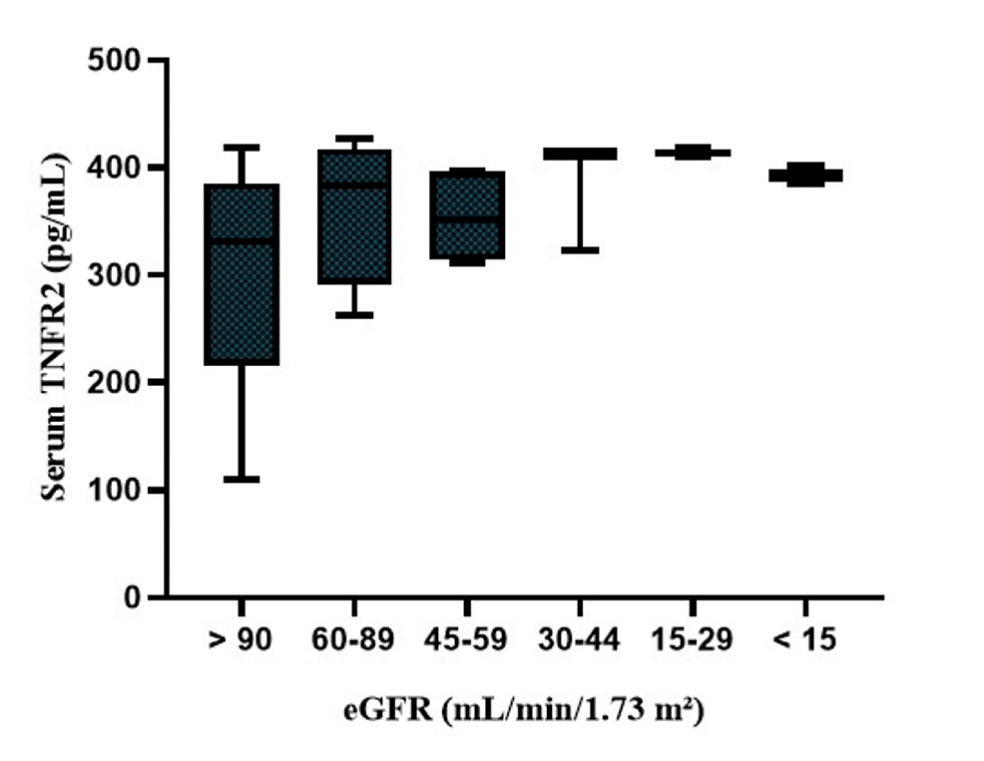
Proportional increase in serum TNFR2 levels with decreased eGFR TNFR2: tumor necrosis factor receptor 2; eGFR: estimated glomerular filtration rate

Following the baseline evaluation, patients were followed up for 18 months. The primary objective was to evaluate the prediction of persistent decline in eGFR <30 mL/min/1.73 m^2^, relapse, and remission. During the median 18-month follow-up period, 19 (38%) patients reached the primary endpoint (consistent decline in eGFR of <30 mL/min/1.73 m^2^ from baseline). We found that patients who had elevated TNFR2 levels had a reduced eGFR (Figure 2).

**Figure 3:**
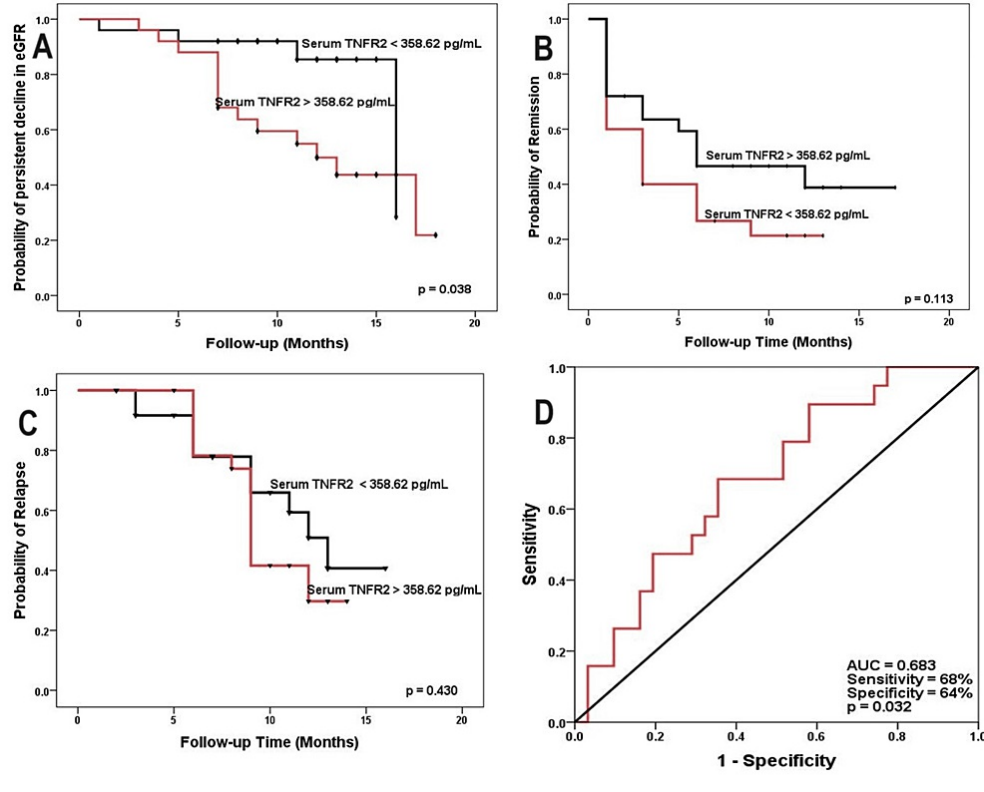
Kaplan-Meier curve for predicting a persistent decline in eGFR <30 mL/min/1.73 m², remission, and relapse stratified by serum TNFR2, and ROC curve for predictive utility for a persistent decline in eGFR <30 ml/kg/min/1.73m² TNFR2: tumor necrosis factor receptor 2; eGFR: estimated glomerular filtration rate; ROC: receiver operating characteristic; AUC: area under the curve

The impact of serum TNFR2 levels on eGFR decline was evaluated using the Kaplan-Meier curve. MGN patients with serum TNFR2 levels below the median level at presentation did not progress to stage 4 CKD on follow-up (p = 0.038). No statistically significant difference was found in terms of remission and relapse in patients with MGN (Figure 3).

In the univariate Cox regression analysis, age (HR: 1.037, 95% CI: 1.00 to 1.07, p = 0.034), serum TNFR2 (HR: 1.008, 95% CI: 1.000 to 1.016, p = 0.050) and serum TNF-α (HR: 1.058, 95% CI: 1.00 to 1.11, p = 0.024) were found to be statistically significant predictors of persistent decline in eGFR <30 ml/kg/min/1.73m^2^. However, after adjusting for other potential confounding variables, only age (HR: 1.051, 95% CI: 1.01 to 1.09, p = 0.006) was found to be a significant predictor in the multivariate analysis (Table 3).

**Table 3:** Cox regression analysis for factors predicting a persistent decline in eGFR <30 mL/min/1.73 m² in patients with MGN MAP: mean arterial pressure; HTN: hypertension; DM: diabetes mellitus; TNFR2: tumor necrosis factor receptor 2; TNF-α: tumor necrosis factor-alpha

	Univariate			Multivariate		
	HR	95% CI	P-value	HR	95% CI	P-value
Age	1.037	1.00 – 1.07	0.034	1.051	1.01 – 1.09	0.006
Sex	0.269	0.06 – 1.17	0.080	-	-	-
MAP	1.010	0.97 – 1.05	0.635	-	-	-
HTN	2.744	0.33 – 22.45	0.347	-	-	-
DM	3.637	1.24 – 10.59	0.018	-	-	-
Serum albumin	0.619	0.34 – 1.09	0.100	-	-	-
Serum calcium	0.963	0.73 – 1.25	0.780	-	-	-
Serum phosphorus	1.201	0.51 – 2.809	0.673	-	-	-
Serum TNFR2	1.008	1.000 – 1.016	0.050	1.008	0.99 – 1.01	0.070
Serum TNF-α	1.058	1.00 – 1.11	0.024	1.052	0.99 – 1.11	0.102

**Figure 4:**
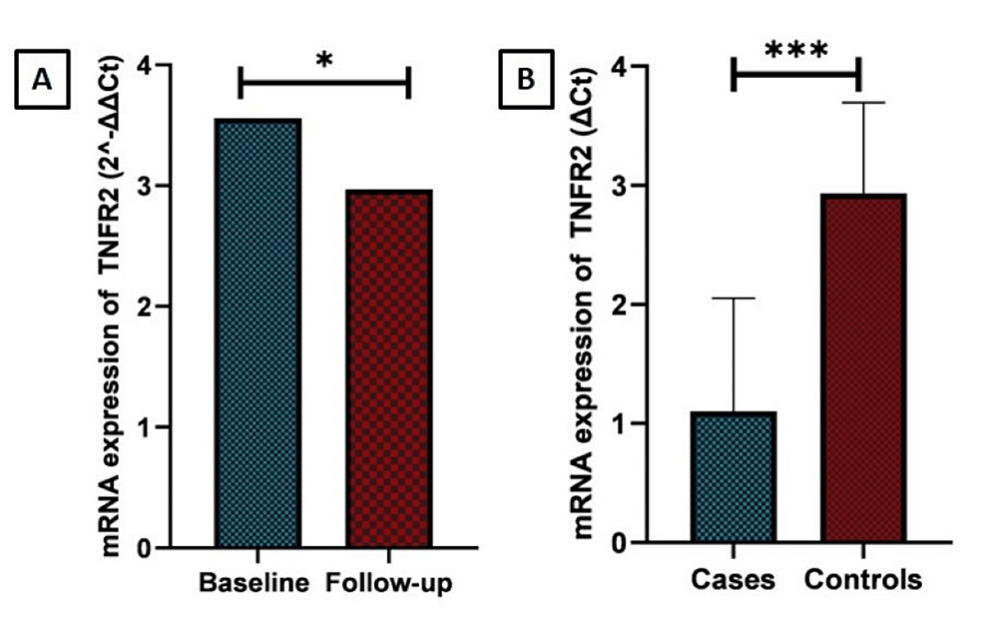
Comparison of TNFR2 gene expression between cases at baseline and follow-up, and between cases and controls TNFR2: tumor necrosis factor receptor 2; mRNA: messenger ribonucleic acid

An ROC curve analysis was performed to calculate the AUC for serum TNFR2 to determine the prognostic ability to predict progression to stage 4 CKD on follow-up. The ROC curve illustrated the prognostic utility of serum TNFR2 in predicting stage 4 CKD, with an AUC of 0.683 (95% CI: 0.534 - 0.831, p = 0.032) and a sensitivity of 68% and specificity of 64%, as shown in Figure 3D. TNFR2 gene expression was upregulated in patients with MGN, with a 3.56-fold increase at baseline and a 2.97-fold increase after follow-up (Figure 4).

**Figure 5:**
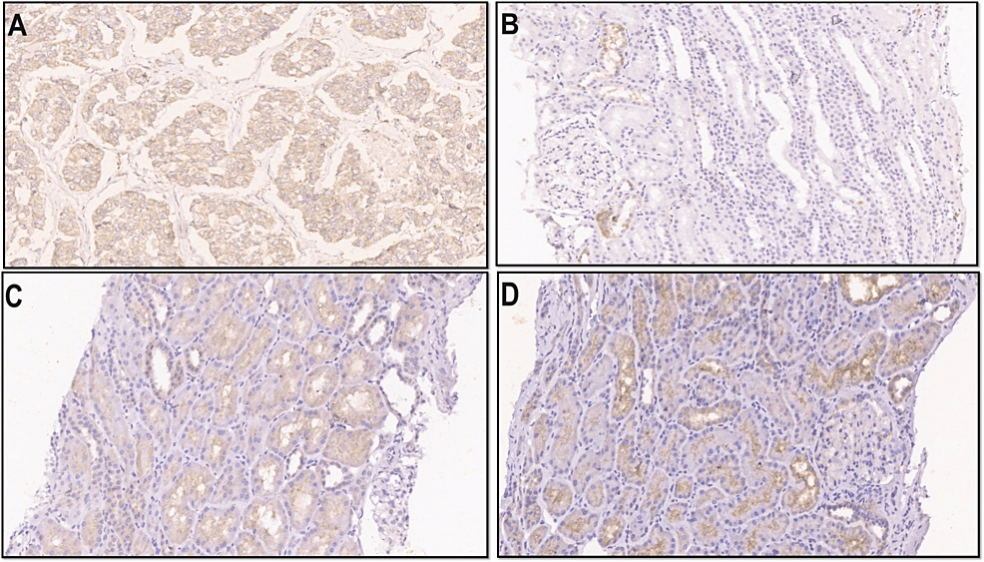
Renal expression of TNFR2 through immunohistochemical (IHC) staining A. Section shows diffuse cytoplasmic expression of TNFR2 in invasive breast carcinoma cells, control stain for TNFR2, rabbit monoclonal antibody from Abcam, and diaminobenzidine stain B. Section shows weak focal expression of TNFR2 in MGN patients in distal convoluted tubules and podocytes. Proximal convoluted tubules are negative. Rabbit monoclonal antibody from Abcam, and diaminobenzidine stain C. Section shows weak focal expression of TNFR2 in proximal and distal convoluted tubules as well as in glomerular podocytes. Rabbit monoclonal antibody from Abcam, and diaminobenzidine stain D. Section shows stronger expression of TNFR2 in proximal and distal convoluted tubules. Rabbit monoclonal antibody from Abcam, and diaminobenzidine stain TNFR2: tumor necrosis factor receptor 2; IHC: immunohistochemistry

TNFR2 gene expression in the cases was statistically higher at baseline and follow-up (p = 0.027) compared to healthy controls (p<0.001). The H-score method was used to evaluate renal TNFR2 expression using immunohistochemistry. TNFR2 expression was assessed in 32 renal biopsies of patients with primary MGN. TNFR2 was expressed in the glomerulus in only two cases, in the PCT in 17 cases with a median H-score of 60 (IQR: 35 - 100) and in the DCT in 16 cases with a median H-score of 45 (IQR: 20 -120) (Figure 5).

There was a statistically significant correlation of serum TNFR2 (r = 0.546, p = 0.023), urine TNFR2 (r = 0.494, p = 0.044), and gene expression of TNFR2 (r = -0.480, p = 0.050) with renal TNFR2 expression in PCT. However, there was no significant correlation of serum TNFR2 (r = 0.401, p = 0.123), urine TNFR2 (r = 0.235, p = 0.381) and TNFR2 gene expression (r = -0.372, p = 0.156) with renal expression of TNFR2 in DCT.

## Discussion

This study delves into the intricate interplay between TNFR2 levels and the decline in eGFR among patients diagnosed with MGN. MGN manifests as autoimmune-mediated damage to podocyte antigens, leading to damage to the glomerular capillary filtration barrier and subsequent proteinuria. Immune complex deposition triggers complement activation and oxidative injury, contributing to the disease progression in MGN [8]. In this study, elevated levels of TNF-α and TNFR2 in patients diagnosed with MGN were compared to those in a healthy control group. We found that serum TNFR2 levels exhibited a negative correlation with eGFR. Individuals with inflammatory kidney conditions like MGN typically display a significant elevation in TNF-α levels, a potent inflammatory cytokine [2]. Lee et al. have found that levels of cTNFRs are notably elevated in patients with idiopathic membranous nephropathy (IMN) compared to individuals with subnephrotic conditions and healthy volunteers [14].

Tonelli et al. identified a correlation between elevated soluble TNFR2 levels and lower kidney function [18]. Elevated levels of TNFR2 have been linked to a decline in renal function, suggesting the involvement of TNF-α inflammation pathway in the progression of the disease. Moreover, there is potential for using TNFR2 as an early indicator of CKD, as significantly higher levels are observed when compared to the control group [7,19]. We found significantly increased serum and urine TNFR2 levels at baseline. The levels of cTNFRs detected during the initial diagnosis were associated with a progressive decline in renal function, indicating that these levels could potentially predict the progression of IMN [14]. Autocrine mechanisms upregulate the production of TNF-α in mesangial cells and podocytes, along with TNFRs, suggesting that an abundance of TNF expression is associated with greater renal dysfunction. Elevated cTNFR levels in the highest tertile correlate with an increased risk of renal failure [14]. In addition, serum TNFR2 was found to have significance in predicting a persistent decline in eGFR via univariate analysis. Studies have suggested that cTNFRs might act as promising biomarkers for monitoring the advancement of CKD in patients with glomerulonephritis, such as IgA nephropathy and MGN [20]. Research conducted at the Joslin Diabetes Center has revealed that high levels of cTNFRs in the blood can accurately predict an early decline in kidney function, leading to progression to CKD3 or end-stage renal disease (ESRD) in individuals with diabetic nephropathy (DN) [13,21].

Diabetes and insulin resistance significantly raise the risk of microvascular complications, including DN, which involves inflammatory processes triggered by hyperglycemia, leading to cytokine production and NF-κB activation, promoting DN development through gene expression of endothelin-1 (ET-1), vascular cell adhesion molecule-1 (VCAM-1), intercellular adhesion molecule-1 (ICAM-1), IL-6, and TNF-α. TNF-α levels are higher in DN cases with microalbuminuria than those without [22]. In DN, mitochondrial energy is disrupted by increased ROS and hyperglycemia, potentially worsening diabetic kidney disease (DKD) if not restored [23,24]. Elevated levels of cTNFR, accompanied by reduced eGFR and elevated proteinuria, are associated with deterioration in renal function and the development of ESRD [20].

We classified serum TNFR2 levels according to CKD stages. Increased serum TNFR2 levels were found to be inversely proportional to eGFR. Lee et al. also demonstrated a proportional worsening of eGFR with an increase in cTNFRs. Additionally, cTNFRs showed a significant negative correlation with eGFR and a positive correlation with proteinuria [14,25]. A study identified TNFR2 as being linked to the onset of CKD with an eGFR of less than 60 ml/min/1.73 m^2^. Moreover, TNFR2 has been found to predict the risk of developing CKD in a cohort of 4926 individuals over a 15-year follow-up period [26]. Conversely, elevated TNFR2 levels are not associated with the accelerated age-related decline in measured GFR (mGFR) [25].

In individuals with MGN with heavy proteinuria, the rate of persistent decline in eGFR and progression to renal failure are expedited. We found proteinuria levels to be significantly elevated in patients who had progressive renal failure, and there is a positive correlation between serum TNFR2 levels and UPCR. In glomerular diseases, extensive damage to the capillary wall allows large pores to form, permitting the passage of high-molecular-weight proteins into the tubular lumen. This overwhelms tubular reabsorption mechanisms, leading to toxic damage and increased urinary excretion of both high- and low-molecular-weight proteins [27]. The presence of TNFRs in the human kidney suggests that soluble forms of these receptors in urine might originate from the exfoliated renal tubular cells [10]. Patients diagnosed with long-standing MGN not attaining remission and having persistent heavy proteinuria are at a higher risk of developing chronic renal failure compared to those with low or no proteinuria [28].

Increased proteinuria may stimulate proximal tubular cells to produce chemokines such as monocyte chemoattractant protein-1 (MCP-1); regulated on activation, normal T cell expressed and secreted (RANTES); and fractalkine, which in turn attract monocytes and T-cells, and IL-8 attracts neutrophils [28]. TNF-α is implicated in causing direct damage to the glomeruli in experimental studies, and this type of injury is significantly decreased in mice that lack TNF-α. Additionally, elevated serum TNF-α levels are linked to the severity of protein leak in DKD, and a reduction in TNF-α appears to coincide with a decrease in urinary protein excretion over time. This may be due to changes in glomerular hemodynamics influenced by lower prostaglandin levels or reduced glomerular permeability. As a result, it seems probable that TNF-α contributes to the progression of nephropathy, especially when there is evidence of protein leakage [18].

TNFR1 is mainly located in endothelial cells of the glomeruli and tubules, while TNFR2 is usually not present in healthy kidneys but the transcription is increased in renal cells during various kidney diseases [14,29]. TNFR1 stimulates the systemic immune response and induces apoptosis of renal T-cells in a model of anti-glomerular basement membrane nephritis, whereas intrinsic cell TNFR2 controls complement-dependent tissue damage [30]. TNFR2 has been detected in glomerular capillaries of renal biopsies from patients with anti-GBM nephritis, and upregulated in allograft rejection [10,31]. We observed a 3.56-fold rise in gene expression levels at the baseline and a 2.97-fold increase during the follow-up along with increased serum TNFR2 levels. We found increased renal expression of TNFR2 in PCT and DCT. Lee et al. showed that individuals with elevated cTNFR levels exhibited greater renal TNFR expression in IHC staining and real-time PCR in patients with MGN, suggesting that the damaged kidney is responsible for the heightened TNFR expression in kidney disease [14]. In this study, the serum and urine levels of TNFR2, along with increased gene expression, exhibited significant differences compared to controls. Also, there was increased immune histochemical expression in the tubules of the kidney. The elevated levels of soluble TNFR2 indicate its potential as a predictor of persistent decline in eGFR. The present study is limited by the relatively smaller number of patients with MGN. Hence, we recommend further studies with larger sample sizes of MGN and longer follow-up periods to validate the predictive potential of TNFR2 levels in serum for wider translational application.

## Conclusions

Elevated TNFR2 levels were associated with renal inflammation and injury, correlating with markers of disease activity. This study highlights the significance of serum TNFR2 as a useful predictive biomarker for eGFR decline in MGN patients, and it can be used to decide on alternate treatment strategies as well as to monitor and predict the progression to ESRD. Furthermore, TNFR2 gene expression analysis and IHC validation highlighted the involvement of TNFR2 in MGN pathogenesis, supporting its potential as a therapeutic target for mitigating renal inflammation and preserving renal function. In addition to its clinical relevance, further exploration of TNFR2-targeted therapies and longitudinal studies investigating the long-term impact of TNFR2 modulation may provide valuable insights into improving outcomes and management strategies for patients with MGN.
